# Improvement of quality of 3D printed objects by elimination of microscopic structural defects in fused deposition modeling

**DOI:** 10.1371/journal.pone.0198370

**Published:** 2018-06-07

**Authors:** Evgeniy G. Gordeev, Alexey S. Galushko, Valentine P. Ananikov

**Affiliations:** N.D. Zelinsky Institute of Organic Chemistry, Russian Academy of Sciences, Moscow, Russia; University of Minnesota Twin Cities, UNITED STATES

## Abstract

Additive manufacturing with fused deposition modeling (FDM) is currently optimized for a wide range of research and commercial applications. The major disadvantage of FDM-created products is their low quality and structural defects (porosity), which impose an obstacle to utilizing them in functional prototyping and direct digital manufacturing of objects intended to contact with gases and liquids. This article describes a simple and efficient approach for assessing the quality of 3D printed objects. Using this approach it was shown that the wall permeability of a printed object depends on its geometric shape and is gradually reduced in a following series: cylinder > cube > pyramid > sphere > cone. Filament feed rate, wall geometry and G-code-defined wall structure were found as primary parameters that influence the quality of 3D-printed products. Optimization of these parameters led to an overall increase in quality and improvement of sealing properties. It was demonstrated that high quality of 3D printed objects can be achieved using routinely available printers and standard filaments.

## Introduction

Additive manufacturing technologies continue to develop extremely rapidly [1–4]. By their principal capacity of reproducing any given geometric complexity they leave all the classical approaches far behind [5,6]; digital manufacturing of goods by means of 3D printing is very fast, cost-effective, and waste-free. Fused deposition modeling (FDM) is the most common technology of 3D printing; it is based on the extrusion and fusion of a plastic filament. This technology is used universally for a huge variety of purposes due to accessibility of personal 3D printing devices and cheap raw materials [7,8].

In recent years, FDM technologies have been intensively employed in direct digital manufacturing applications to facilitate obtaining of full-fledged final products. This trend is supported by both the widespread use of personal 3D printers and the increase in the range of materials for 3D printing. Online 3D printing services also promote to popularization and distribution of 3D printing [9]. The key advantage of such services is possibility for printing objects from various materials by a wide range of technologies many of which are not yet available for personal using, for example selective laser sintering of powder materials. When using online 3D printing services, the user uploads his 3D model to the service server, selects the material for manufacturing, and at the end obtains the printed product. To select the most optimal for the customer online 3D printing service, one can use the aggregator service, which contains information on the additive manufacturing services available in different regions and the appropriate 3D printing technologies [10]. If the user is a beginner in the 3D modeling, today there are many databases of ready-made 3D models that can be downloaded for further learning [11].

Thermoplastic polymers available to date include acrylonitrile butadiene styrene (ABS), polylactide (PLA), nylon, polyethylene terephthalate glycol-modified (PETG), polycarbonate (PC), polyoxymethylene (POM), polyethylene (PE), high impact polystyrene (HIPS), polyvinyl alcohol (PVA), and, in particular, a number of chemically and thermally stable materials such as polypropylene (PP), polyether ether ketone (PEEK), polyetherimide (ULTEM), and polyphenylsulfone (PPSU) [12,13]. These materials can be modified with various fillers: carbon fibers, fiberglass, metal particles etc., which further extends and enhances the scope of FDM application [14–17].

Important to note, FDM technologies are increasingly used for the design of scientific and industrial equipment, and the number of studies related to this topic is growing continuously. These technologies have been proved to be very relevant for the purposes of biochemistry and medicine [18–24], analytical equipment engineering [25–30], biotechnologies [31,32], pharmaceutics and fine organic synthesis [33–38], as well as in the designing of chemical reactors [39–42] and invention of new materials for electronics and energetics [43–45].

However, the popularity of FDM has brought to the forefront the critical limitation and major inconsistency of this technology, manifested as an insufficient quality of the printed items due to their structural defects (high porosity) and unsatisfactory sealing properties. Effective sealing of gases and liquids is a necessary requirement for the design of devices used in research, industry and a variety of practical applications [46].

The present article describes a new approach for assessing the quality of 3D printed objects. We show that optimization of certain 3D printing parameters can bring a substantial improvement in the quality of FDM-created products. We also evaluate the influence of key printing parameters on the quality of resulting FDM-products. An additional experimental confirmation of the obtained data is provided by a comparative study of a highly complex chemical process (C-C coupling process applied in pharmaceutical industry [47,48]) in a series of 3D printed chemical reactors.

## Results and discussion

**Fig 1:**
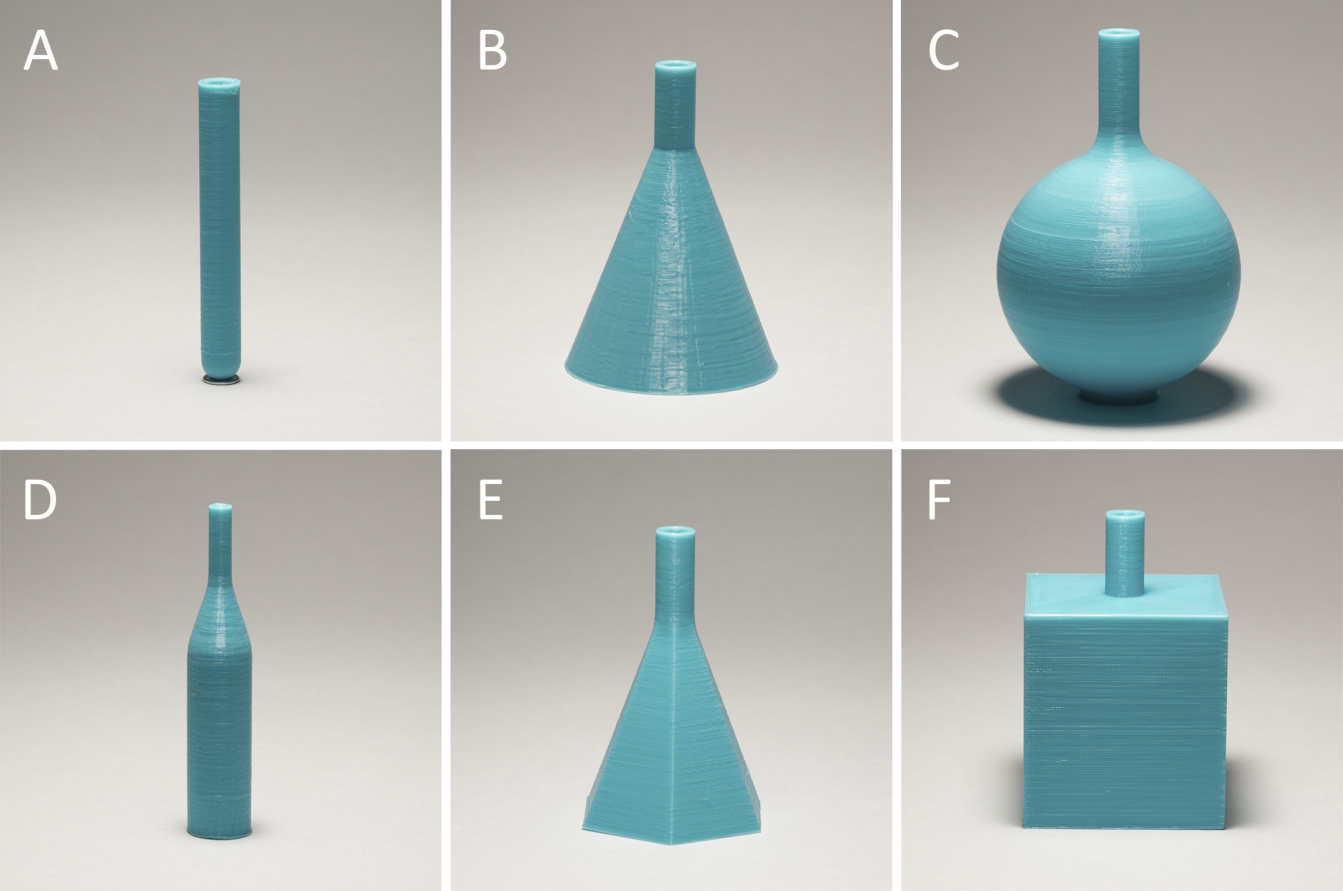
Objects of various shapes made of PLA by 3D printing. (A) cylinder, (B) cone, (C) sphere, (D) joined cylinder/cone, (E) pyramid, (F) cube.

Objects of various shapes were produced using a FDM technology based on the molten thermoplastic polymer extrusion through a metal nozzle with an opening diameter of 0.30 mm. Multiple layers of the product were created by sequential addition of the plastic filaments, which fused with one another and provided a volume increment. Due to the small extrusion nozzle opening diameter and effective fusion of the layers, the products had relatively smooth surfaces and satisfactory mechanical strengths. All basic geometric shapes were produced and analyzed (Fig 1): cylindrical (A), conical (B), spherical (C), joined cylindrical/conical (D), pyramidal (E) and cuboidal (F). The items were printed from a PLA filament using a personal desktop 3D printer.

**Fig 2:**
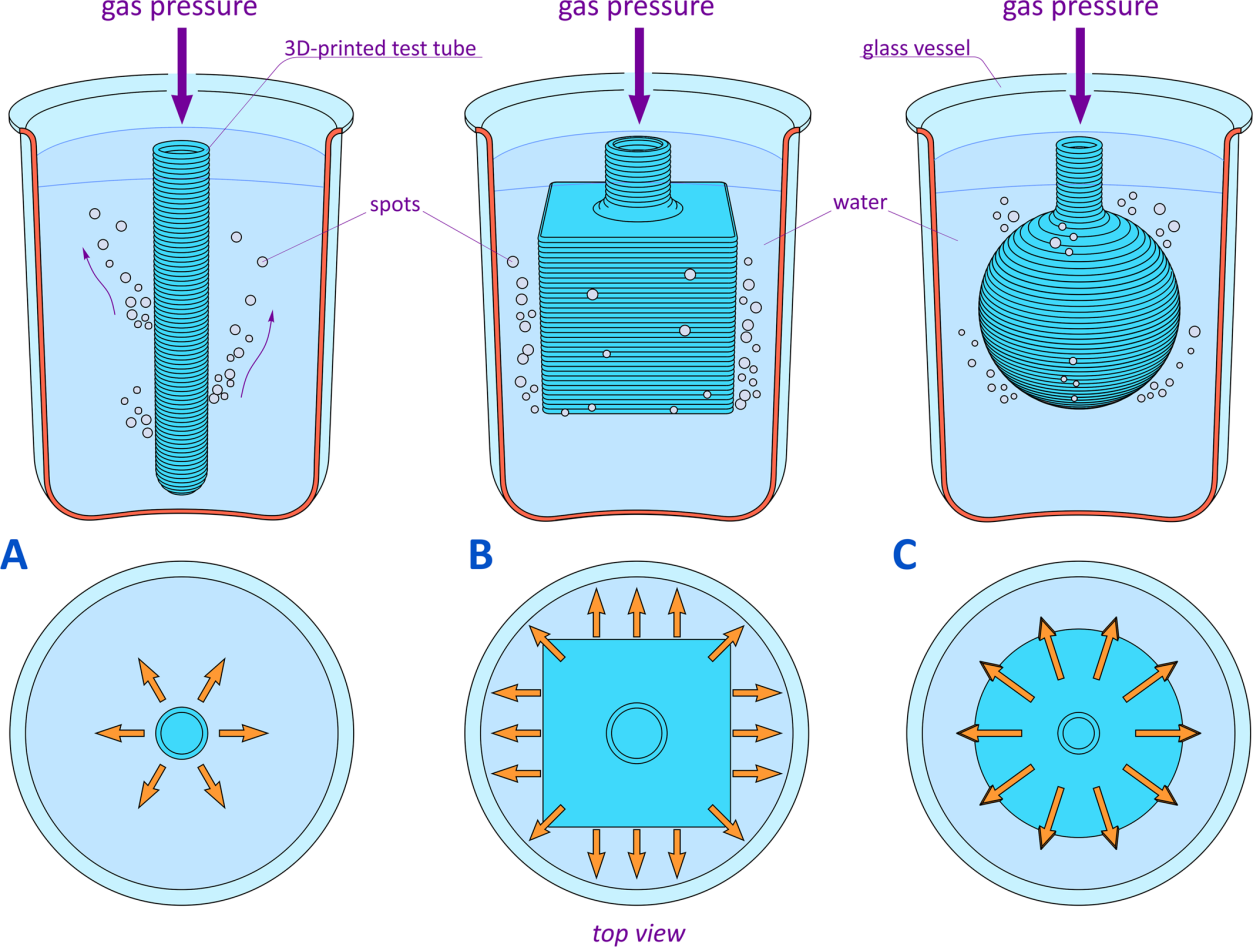
Evaluation of quality of 3D-printed shapes. (A) cylindrical test tube, (B) hollow entity of cubical shape, (C) spherical flask. In the top view, arrows indicate outgassing.

To evaluate the quality of the printed products, we developed the following experiment (Fig 2). During the test, the printed objects were connected to an air compressor by means of a flexible pipe and were placed in a transparent glass container filled with water. A slight internal gas pressure applied through the pipe induced outgassing in the form of bubbles emanating from the intrinsic pores, which permeated the wall of the product. The intensities and densities of the bubble flows corresponded to the linear dimensions and densities of the pores, respectively. The larger the diameter of the pore, the more intense the formation of air bubbles through this pore. The quantitative density of air bubbles on the surface of the printed part corresponds to the density of the through channels inside the wall. This experiment provided both visualization and quantitative assessment of the 3D printing quality. Importantly, the described approach was applicable to the objects independently of their shape (Fig 2).

**Fig 3:**
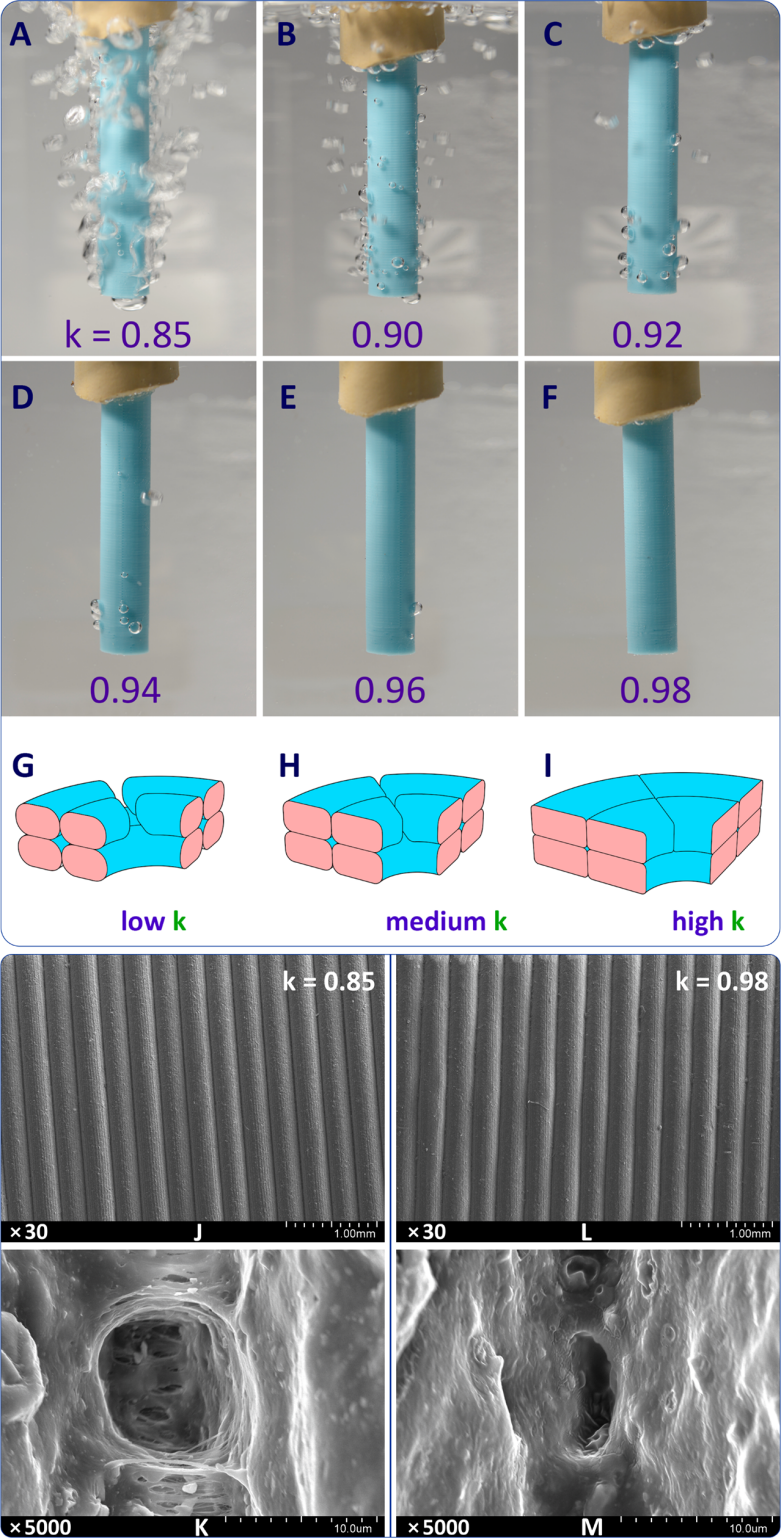
Experimental demonstration of sealing properties of 3D-printed tube at different k-values. (A-F)–Evaluation of the of the number of pores in the wall of the tubes manufactured at different k-values; (G-I)–schematic representation of the effect of k-value on the porosity; (J, K)–electron microscopy image of the outer surface of an object printed at k = 0.85 showing a perforating pore (K); (L, M)—electron microscopy image of the outer surface of an object printed at k = 0.98 showing a smaller pore filled with polymer (M).

The experimental implementation of this approach, including the device assembly (see Supporting Information for an image of the device), was relatively easy and technically uncomplicated. The first 3D printing quality test was carried out for a cylindrical object (Fig 1A). The buildup of gas pressure inside the object by an air compressor led to an intensive release of gas, clearly detectable upon immersion of the object in water (Fig 3A). The observed effect was consistent with scanning electron microscopy data: although at the mm-scale the object was composed of even polymer layers (Fig 3J and 3L), it contained numerous pores clearly discernible at higher magnifications (Fig 3K and 3M). The average size of these pores was 10–20 μm; arranged chaotically, they created a defective structure heavily compromising the sealing properties and impairing the overall quality of the material.

By varying several 3D printing parameters in different combinations, it was shown that the porosity of FDM-products depended most strongly on the extrusion multiplier (k-value). Extrusion multiplier is the parameter for controlling extrusion flow rate, i.e. the volume of melted plastic material extruded through the nozzle per unit time. Technically an increase in the extrusion multiplier usually leads to an increase in the speed of rotation of the gears in the feeding mechanism of the printed head.

The items printed at low k-values (k = 0.85) were highly porous (Fig 3A and S8 Fig). 3D printing at increasingly higher k-values led to a gradual reduction in the porosity, together with a reduction in the pressure-forced outgassing (Fig 3A and 3F). At k = 0.98 the release of gases through the walls of an object was completely blocked, indicating the effective sealing and overall improvement of the material quality. To quantify the effect of the extrusion multiplier on the impermeability of FDM-vessels, we measured the volumetric flow rate of air passing through the porous wall of the vessels (S10 Fig). For different values of the k, the volumetric flow rate is 24.0; 10.0; 1.0; 1.5; 0.0; 0.0 ml/s for k = 0.85; 0.90; 0.92; 0.94; 0.96; 0.98 correspondingly. Thus, the impermeability of the vessels is very sensitive to a change in the extrusion multiplier value: a change in k by only 5% leads to a decrease in the volumetric flow rate by more than two times, and a further increase in k leads to the fully impermeable product.

Scanning electron microscopy examination showed much fewer defects and the absence of perforating pores (Fig 3L and 3M and S5 and S6 Figs).

In order to understand the mechanism of the observed improvements, it should be taken into account that the extrusion multiplier reflects the amount of the molten polymer extruded from the nozzle per time unit. At low k-values, a specific combination of velocity, viscosity, pressure, etc. of the molten material favors the appearance of characteristic voids within the layered structure and, ultimately, the emergence of perforating pores (Fig 3G). As k increases to medium values, the number of voids in the material decreases and the pore diameter also decreases (Fig 3H). The highest quality of 3D printing and, in particular, good sealing performances are achieved at high k-values (Fig 3I and S8 Fig).

All the tested k-values within the range of 0.8 to 1.0 produced no noticeable changes in the surface appearance: an increase of k within this range resulted in an improved quality and enhanced sealing properties of the products without negative side effects. This trend (an increase in the k-value leads to a decrease in the porosity) is apparently reproduced regardless of specific technical details of printing, though exact effective ranges of the k-value can vary depending on the feeder construction, printer model, and type of the material. However, an excessive increase in the k-value (k > 1) in our setting resulted in visible surface defects in the form of undulations of the surplus material (Figure F in S8 Fig).

3D printing at k = 0.98 invariably yielded products with satisfactory sealing properties for all the tested materials, which included PLA, ABS, Nylon, Nylon-C (carbon-fiber reinforced nylon), PETG, and PP (S1 Fig). Changing the extrusion temperature within the range of 180–220°C at k = 0.98 had no substantial effect on the sealing properties of the product (S2 Fig).

These results were reproduced using another 3D printer, for example, Designer X Pro. This printer has a modified extruder design and a modified way of controlling the k value (by changing the profiles of plastics, without using a slice program). A k value equal 0.85 resulted in permeable tube with a porous internal wall structure, whereas k = 1.2 leads to an impermeable product with a solid wall (S8 Fig). However, increasing k to 1.2 impair the quality of the outer surface of the printed item, leading to roughness in some places. For the Designer X Pro printer, it was possible to obtain an impermeable product at slightly higher k values than for the Picaso 250 Designer Pro printer, because of these devices differ from each other in the design of extruders.

**Fig 4:**
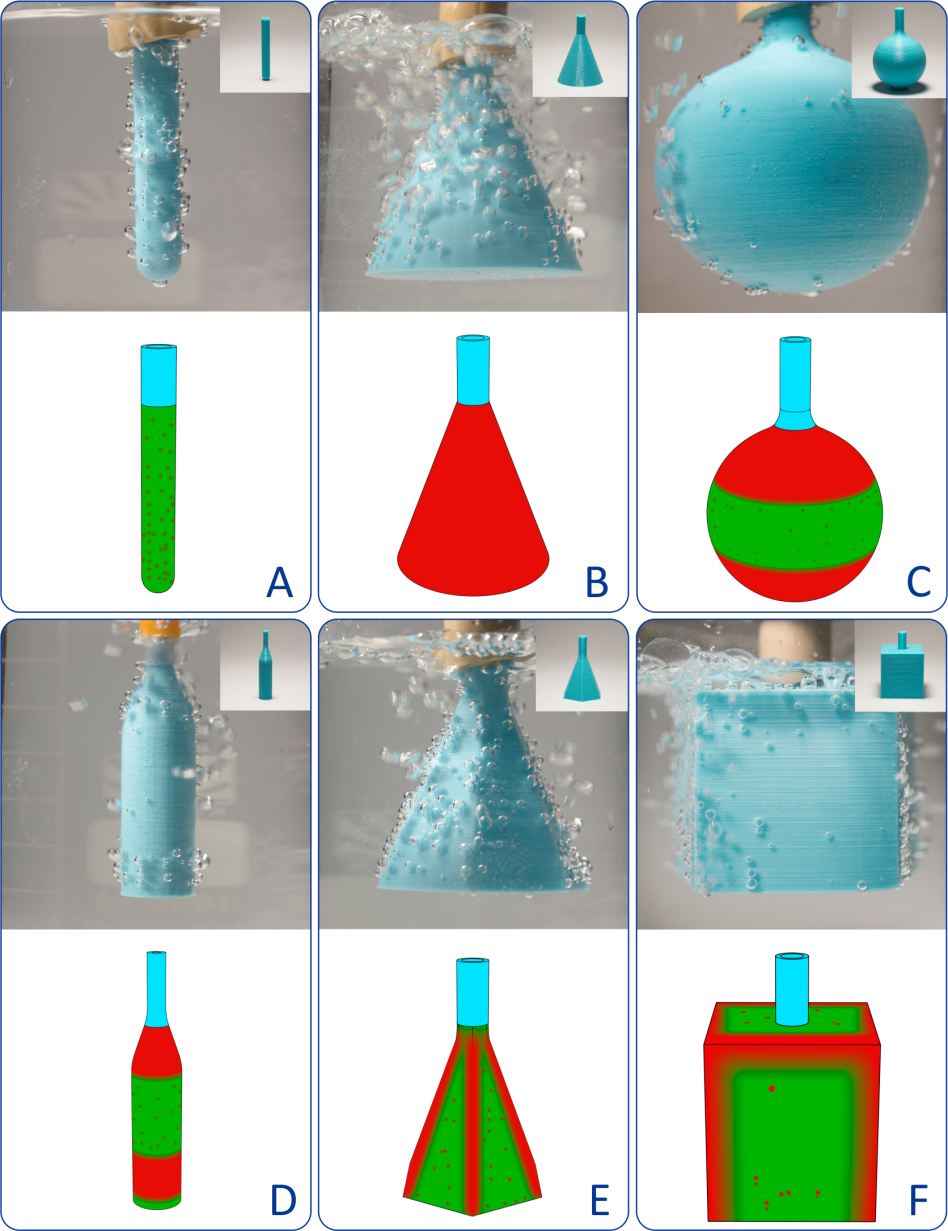
Functional assessment of 3D printing quality for objects of different shapes. All objects are printed with identical parameters at k = 0.9 from PLA: (A) cylinder, (B) cone, (C) sphere, (D) compound shape, (E) pyramid, (F) cube. The diagrams below show the distribution/densities of the pores. Red areas have maximal porosity/permeability; green areas are relatively impermeable; blue color designates junctions with the air compressor.

One of the most important advantages of 3D printing is the possibility of creating objects of an arbitrarily given shape; therefore, in the context of this study, the dependence of the properties of the product on its geometric shape is of particular interest. In this regard, we printed and tested all the basic geometric shapes (Fig 4). It turned out that under standard conditions cylindrical objects had the minimum number of pores and the best quality of 3D printing (Fig 4A). Conical objects had larger pores uniformly distributed over the surface (Fig 4B). For a spherical shape, the largest number of pores was observed in the region of poles lying on the axis perpendicular to the planes of the layers, while the equatorial region retained impermeability (Fig 4C). A combination of cylindrical and conical shapes in one object resulted in a uniform distribution of a small number of pores in the wall of the cylindrical portion, and a much larger number of pores in the conical portion (Fig 4D). In plane-faced products (e.g. hexagonal pyramid and cube), the most porous areas were found at the edges, that was, in the neighborhood of the joints between the faces (Fig 4E and 4F). The observed dependence of porosity on the geometric shape or its specific area is explained by the corresponding differences in the mode of layer positioning: in cubical and cylindrical products, the layers are arranged exactly one above the other, so the interlayer contact is most effective. In conical products, the layers are arranged with a certain offset, that is, stepwise, which makes the interlayer contact less effective. The dependence of the porosity on the effectiveness of interlayer contacts is clearly illustrated by the overall distribution of pores in a spherical product: in the polar regions (Fig 4C), the layers are arranged with displacement, while the equatorial region is topologically close to the cylinder–here the layers are stacked almost exactly one above the other, and because of this the number of pores decreases.

Fig 4 shows the distribution of bubbles in the "dynamic" mode, that is, in the process of their formation when excessive internal pressure. In this mode, floating up bubbles are superimposed on the wall of the vessel and the pore localization points cannot always be identified unambiguously. A more clear representation of the pore localization points is given by the distribution of bubbles in the "static" mode (S9 Fig), that is, after the internal pressure is dropped down to standard conditions. In the "static" mode, the bubbles do not float up, but remain at the places of their formation, therefore the pores can be localized unambiguously.

The experiments show that edges and vertices are more prone to defects than other areas. Among the shapes with smooth outlines, conical and spherical elements of irregular curvature are most vulnerable, whereas flat and cylindrical surface areas are most resistant to the pore formation.

**Fig 5:**
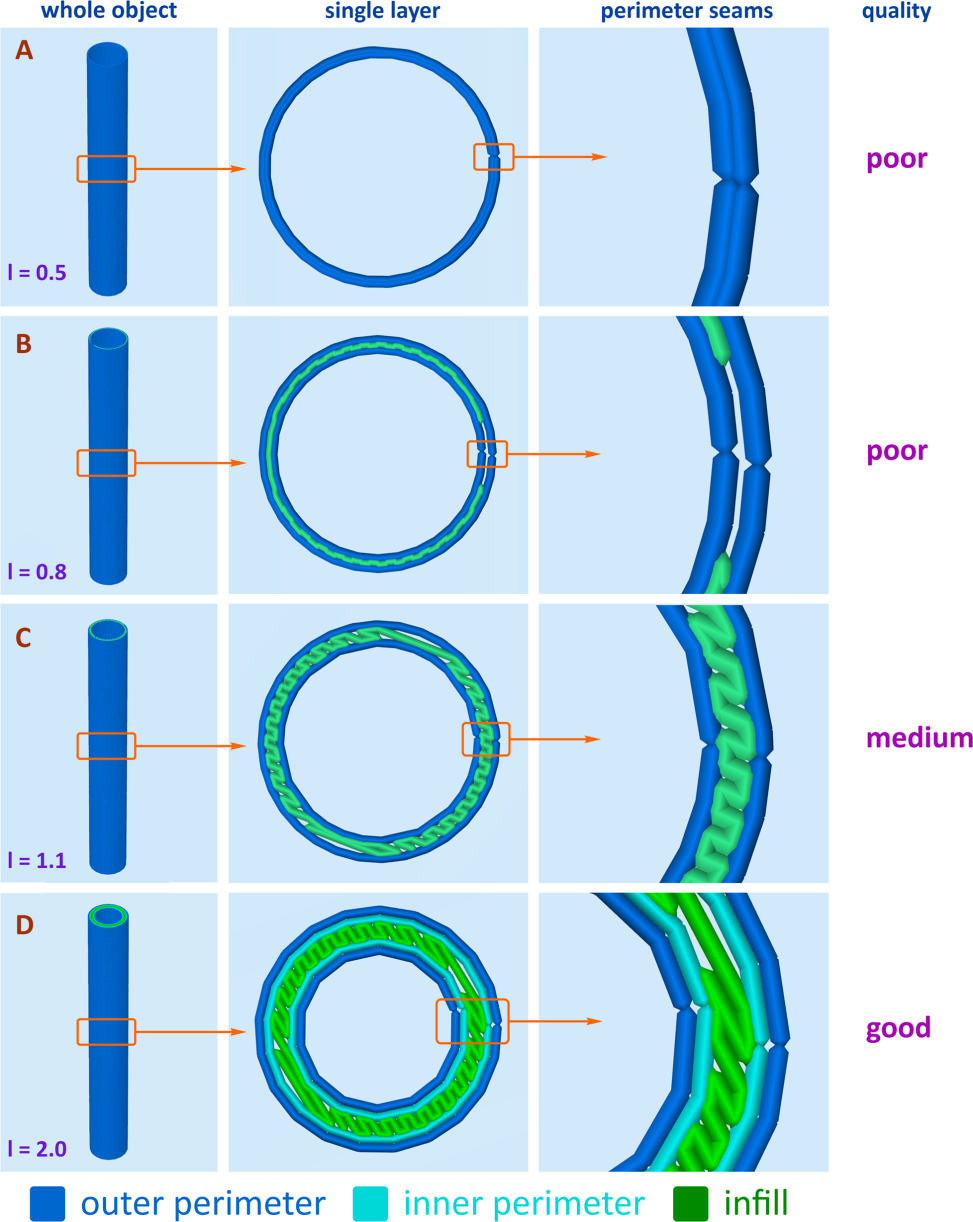
Printed cylindrical tubes of different wall thickness (l, mm). For each l value the corresponding wall structure and printing quality are displayed (all items were printed at k = 0.98).

In a number of cases, the selection of the optimal k-value turns out to be insufficient to ensure effective sealing. A simple increase in the product wall thickness at a fixed k-value does not work in such cases. The solution can be achieved by adjusting the toolpath information for 3D printing generated by slicer software (G-code). The G-code determines the exact positioning of plastic layers during the creation of a required shape by 3D printing. The type and mode of the infill, also programmed by the G-code, are set in accordance with the required wall thickness and the number of inner and outer perimeters. To evaluate the importance of the G-code, we examined four different types of plastic wall formation (Fig 5 and S3 Fig). As can be seen from the G-code, there are no branched channels in the walls of the printed products, that is, each pore corresponds to one through channel. All items in these series were printed at k = 0.98; the results are as follows.

In thin-walled printed products, the entire product wall is composed of concentric contours (outer perimeters) with no room for infill (Fig 5A). When the discontinuous sites (seams) of the perimeters coincide, a perforating pore appears. Indeed, the items printed this way (which provides the wall thickness of about 0.5 mm) are often completely untenable in terms of sealing, for all the possible k-values.

When the wall thickness is set up at about 0.8 mm, the slicing software prescribes another way of 3D printing, because the space between the arrays of outer perimeters is no longer equal to zero. This space is filled with randomly oriented segments of the polymer, which constitute the primordial infill. However, since this space is quite small, its filling is uneven, with the formation of numerous voids (Fig 5B). If such a void appears at the site where the seams of the perimeters coincide, a perforating pore appears. However, the probability of such an event is lower than the probability of the coincidence of seams in the complete absence of infill, hence the decrease in the pore number.

At greater wall thicknesses (1.1 mm and 2.0 mm, corresponding to sequential discrete inner perimeters) the internal space of the wall is large enough to ensure the homogeneous structure of the infill (Fig 5C and 5D). Therefore, by using these values of thickness with appropriate G-codes, normally generated automatically by the slicer software, the pore numbers are reduced to a minimum. The sealing properties of 3D printed products with wall thicknesses of more than 2 mm can be further corrected by changing the 3D printing parameters *per se*, primarily the k-value.

Thus, to minimize the porosity, the proper filling of the inner space should be additionally controlled by verification of the G-code suggested by the slicer software. The more homogeneous the intermediate layer of the wall is, the more impermeable the wall of the product will be, since all the seams will be securely insulated from each other.

Since FDM technologies have no limitations on the geometric complexity, we manufactured containers of various compound shapes, combining flat-faced and cylindrical portions (S4 Fig). Despite the optimization of the k-value, which substantially contributed to an improvement in the compartment-isolating properties of the products, the complete sealing was not achieved: non-cylindrical portions of the printed items, especially the edges and conical segments, invariably contained perforating pores. Thus, the cylindrical shape was most favorable for creating products with a thin impermeable wall using commercial personal 3D printers.

**Fig 6:**
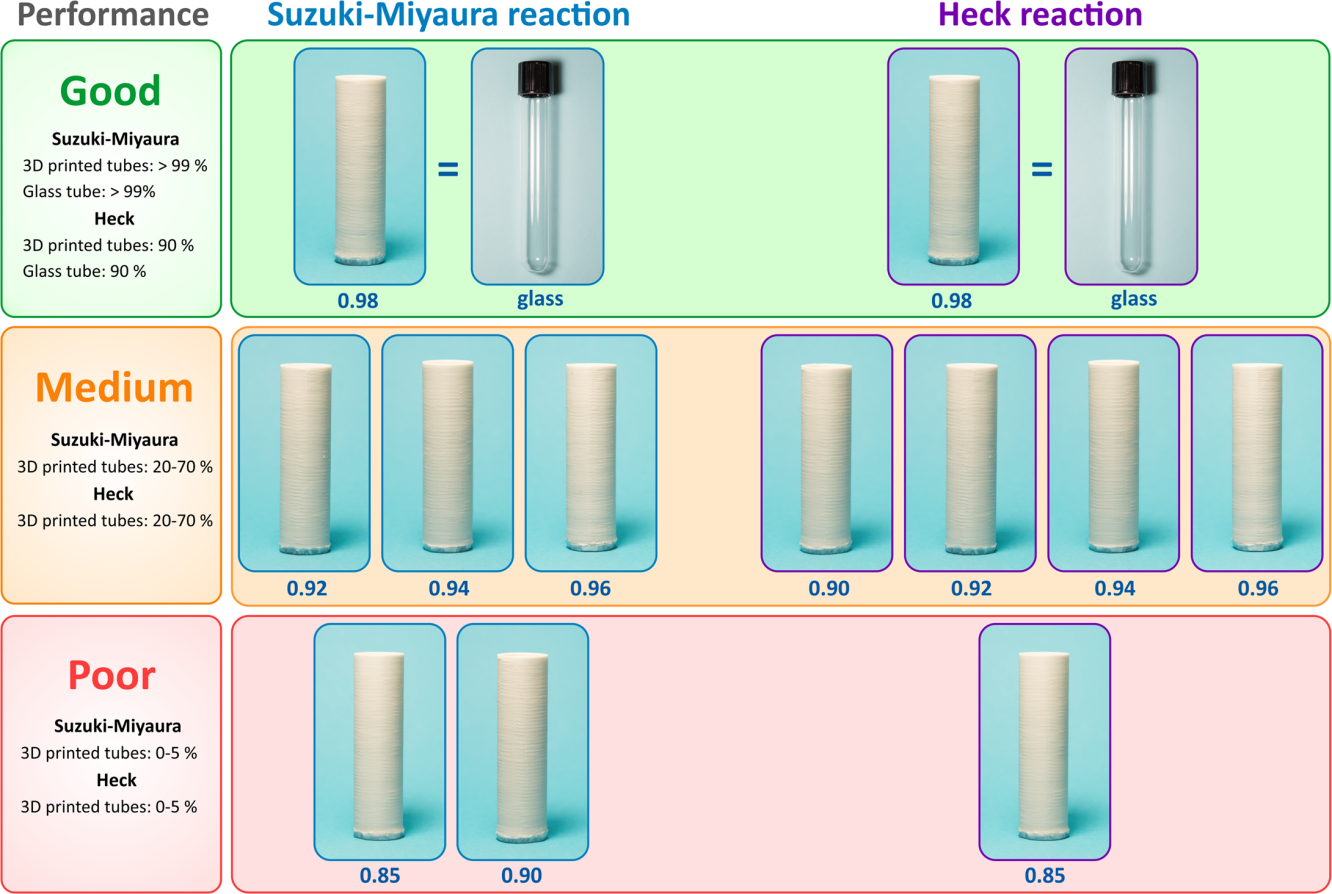
Operational reliability of 3D printed polypropylene tubes. PP tubes as chemical reaction vessels in comparison with conventional glass test tubes. Values of k are given below for each 3D-printed tube. Performance in the studied chemical transformation is illustrated by product yield (in %) in each studied case, where ≥ 90% efficiency corresponds to excellent performance.

The 3D printed polypropylene tubes were subjected to a challenging experimental test for operational reliability. The tubes were used as reaction vessels for the catalytic Suzuki-Miyaura and Heck chemical transformations (currently, most demanding C-C coupling processes used in pharmaceutical industry). The Suzuki-Miyaura reaction was performed with 1-bromo-4-nitrobenzene and phenylboronic acid as coupling partners and potassium carbonate as a base, in ethanol/water at +70°C for 5 h. The Heck reaction between 1-bromo-4-nitrobenzene and styrene with triethylamine as a base was carried out in N-methyl-2-pyrrolidone at +140°C for 6 h. Both reactions were catalyzed by a 1% Pd/C palladium on carbon catalyst; conversion rates were measured using NMR spectroscopy. Conventional glass test tubes were used for comparison and a summary of the results is illustrated in Fig 6.

Poor performance not suitable to carry out the Suzuki-Miyaura reaction was found for the test tubes printed with k-values in the range of 0.85–0.90 (Fig 6). The reaction could be carried out in 3D printed test tubes of a better quality (k = 0.92–0.96), although lower performance was observed as compared to the glass tubes. The quality assessment described above showed that structural defects were eliminated at k = 0.98. Indeed, the Suzuki-Miyaura reaction in an ethanol/water solvent was performed with excellent performance totally comparable with that in the glass tubes (Fig 6). Similar effects were observed when using the 3D printed polypropylene tubes under more stringent conditions of the Heck reaction. Tubes printed at relatively low k-values (0.85–0.96) could show compromised sealing properties at the preparatory stage or during the reaction itself, whereas the walls of a tube printed at k = 0.98 were impermeable, which allowed carrying out the Heck reaction with the same conversion rates as in a conventional glass test tube (Fig 6).

## Conclusions

In this paper, we describe a convenient approach to assessing the quality of 3D printed objects. The wall permeability of 3D printed objects strongly depended on its geometric shape and gradually decreased in the following order: cylinder > cube > pyramid > sphere > cone. It was found that neither the extrusion temperature, nor the type of the polymer substantially influenced the quality (porosity).

By using the proposed approach, it was shown that the basic parameters influencing the porosity of 3D-printed products were the extrusion multiplier, wall thickness and G-code. Optimization of these parameters led to an overall increase in the quality and an improvement of sealing properties.

To manufacture impermeable items, one should take into account the following observations:

- usage of larger extrusion multiplier value may be useful (sometimes as large as possible), unless this results in a significant deterioration in the surface quality of the item;
- the wall thickness should allow for an internal diagonal filling;
- it is desirable to distribute the seams for each layer not in one line, but in random positions, although this is not an indispensable requirement;
- among geometries studied here, cylindrical surfaces are preferred whenever possible for easier optimization to the level of improved defect-free object.

The impermeability of FDM vessels is very sensitive to extrusion multiplier variation: a slight change in k value may lead to a significant improvement in the impermeability parameter.

Polypropylene items printed with the optimized parameters were impermeable and resistant to the stringent conditions required for the Suzuki-Miyaura and Heck reactions. Conversion rates of the reactions carried out in 3D printed tubes of this type were the same as for conventional glass test tubes. Thus, 3D-printed objects composed of chemically stable materials can be suitable for manufacturing of laboratory equipment.

The present study shows that 3D printing is suitable for the production of completed and functionally consistent products with good sealing properties from a wide range of polymers even by using inexpensive personal 3D printers. Of course, each specific case of manufacturing by 3D printing can require a separate optimization of parameters, especially when changing the model of a 3D printer or filament material. The product properties can be affected by the feeder construction, presence/absence of a closed case, heating mode of the working platform, extruder cooling system, etc. Despite that, with proper optimization of printing conditions, commercial desktop 3D printers can be suitable for the production of sealed containers for various applications. The proposed quality assessment procedure allows the gradual improvement of the quality of 3D printed objects by elimination of structural defects.

## Experimental

### General materials and methods

All items were manufactured using a personal desktop Picaso 250 Designer Pro 3D printer (PICASO 3D) with a 0.3 mm diameter nozzle. Processing of a digital 3D model (G-code generation) and setting up of the manufacturing process were carried out using the Simplify3D printing software (Simplify3D 3.1.1, Simplify3D, LLC, 2016). Some vessels were manufactured by Designer X Pro printer for comparative analysis.

All 3D models were created by NURBS modeling.

For 3D-printing, several thermoplastic materials were used: colored PLA—polylactide (Esun), colored ABS—acrylonitrile butadiene styrene (Esun), transparent PETG—polyethylene terephthalate glycol-modified (FL-33), polypropylene PP—(FL-33), Taulman 645 Nylon (taulman3D), carbon filled Nylon (Fiber Force). It should be noted that during the printing with carbon-filled filaments, it is recommended to use a 0.4–0.6 mm diameter nozzle, since a standard 0.3 mm diameter nozzle can lead to unstable printing due to clogging of the nozzle with the filled polymer. In addition, a natural (unfilled) nylon filament can slip in the guide wheels of an extruder with a direct feeding mechanism which interrupts the plastic extrusion. Therefore, printing by the natural nylon filament is much more convenient if there is a mechanism for adjusting the clamping force for pushing the filament. Carbon filled nylon is much more favorable for FDM printing, because such material has a rough surface and does not slip in the feeding mechanism of the extruder. For printing by ABS and Nylon, a heated 3D printer bed is required. In addition, to improve the adhesion of the part to the bed, a special water-soluble Cube Glue adhesive (3D Systems, Inc.) was applied for bed surface coating. It should be mentioned that polypropylene parts have poor adhesion even to the special adhesive; therefore, PP parts were printed on a 2 mm thick PP sheet.

### 3D printing with standard parameters

General printing parameters used for all products were as follows:

Layer High 0.3 mm; Extrusion Width 0.30–0.36 mm; Retraction Distance 1.0 mm; Extra Restart Distance -0.2 mm; Retraction Vertical Lift 0.4 mm; Use random start points; Infill 100%; Outline Overlap 50%; Outline Direction Inside-Out; Printing Speed 2400 mm/min.

Items of various shapes (Figure A in S7 Fig) were printed from PLA at k = 0.9, PLA extrusion temperature (T_ext_) 220°C, heated bed temperature (T_bed_) 50°C, and cooling 60%.

Manufacturing of PLA tubes for the assessment of the feed rate influence on the permeability (Figure B in S7 Fig) was carried out at T_ext_ = 220°C и T_bed_ = 50°C.

Manufacturing of PLA tubes at different T_ext_ (Figure C in S7 Fig and S2 Fig) was carried out at k = 0.98 and T_bed_ = 50°C.

Manufacturing of PLA tubes of different wall thicknesses (S3 Fig) was carried out at k = 0.98 and T_bed_ = 50°C.

Manufacturing of tubes from different materials (Figure E in S7 Fig and S1 Fig) was carried out at k = 0.98 and printing speed 2400 mm/min; the other conditions were as follows:

PLA: T_ext_ = 220°C, T_bed_ = 50°C, cooling 60%;

ABS: T_ext_ = 230°C, T_bed_ = 100°C, cooling 10%;

PETG: T_ext_ = 230°C, T_bed_ = 70°C, cooling 10%;

PP: T_ext_ = 235°C, T_bed_ = 80°C, cooling: none;

Nylon: T_ext_ = 245°C, T_bed_ = 100°C, cooling: none;

Nylon-C: T_ext_ = 245°C, T_bed_ = 100°C, cooling: none.

### Assessment of quality of 3D-printed objects

For permeability testing, FDM products were immersed in a glass filled with water. For connection with the compressor, all parts were equipped by pipes. Permeability tests were carried out using an air compressor (inside excessive air pressure was about 0.5 bar). The connection to the compressor was realized using a vacuum hosepipe. Excessive internal pressure resulted in air bubbles seeping through the pores in the part wall. The air bubbles were detected visually. The amount of pores in the wall of the part was proportional to the number of bubble flows. The pore size was approximately proportional to the intensity of the bubble flow.

Measurements of the volumetric flow rate of air passing through the vessel wall were carried out by glass graduated cylinder filled with water and immersed with an open end into a container with water (S10 Fig). The FDM vessel connected to the compressor was placed inside the graduated cylinder and the volume of air that passed through the wall was equal to the volume of water displaced from the cylinder. The time of the water displacement was measured using a stopwatch. The internal air pressure for these measurements was equal to 4 bars.

As a real-world stress test, the Suzuki-Miyaura and Heck reactions were carried out in PP test-tubes since PP was the most chemically resistant material. The PP tubes were stable towards N-methyl-2-pyrrolidon and remained sealed during 6 hours at 140°C with stirring.

### Optimization of 3D-printing parameters

The permeability of 3D-printed objects can depend on a variety of parameters: the extrusion multiplier, wall thickness, type of the internal filling of the walls, extrusion temperature, thermoplastic type, and shape of the part. These parameters were optimized to achieve the desired permeability of 3D-printed objects.

The extrusion multiplier (k) varied in the range of 0.85–1.00 for Picaso 250 Designer Pro printer and 0.85; 1.2 for the Designer X Pro printer. The increase in k leads to an increase in the volumetric flow rate of plastic extrusion. This leads to filling of small pores by plastic material in the part wall. However, too great k value causes the appearance of defects on the product surface due to material "overflow". Therefore, the extrusion multiplier should be fine tuned for selection of some optimum value to achieve impermeability on one hand and a high surface quality on the other hand. The selection of k value is usually performed when preparing the model for printing in the slicer program (for the Picaso 250 Designer Pro printer) or when setting the profile for each plastic (for the Designer X Pro printer).

The wall thickness varied within 0.50–2.00 mm. Variation the thickness of the wall allows to vary the number of external and internal perimeters, as well as the volume of the internal space of the wall, designed for diagonal filling (Fig 5). Too small wall thickness results in a lack of diagonal filling and high porosity of the part. The wall thickness should be sufficient to allow internal infill, which significantly improves the impermeability of the product. Varying the wall thickness is carried out at the design stage of the model in the CAD program.

The temperature of extrusion varied in the range of 180–220°C for PLA. An increase in the extrusion temperature leads to a greater fluidity of the polymer melt. The high fluidity of the melt provides a more efficient filling of the pores in the wall of the part. However, an excessive increase in the extrusion temperature can lead to the decomposition of the polymer, the formation of a polymer jamming in the nozzle of the extruder.

Various thermoplastic polymers were tested: PLA, ABS, PETG, PP, Nylon, and carbon-filled Nylon. Different materials have different interlayer adhesion. However, in this work, the variation of the materials did not have a significant effect on the impermeability of the part. If the temperature parameters of the printing are optimal for specific material, the part made of any material are sufficiently sealed.

To investigate the effect of the shape of the FDM parts on the permeability, the shape of a pyramid, cube, sphere, cone, cylinder, and complex compound forms were tested.

The combination of such parameters as the wall thickness, nozzle diameter and number of internal and external perimeters determines the character of the internal filling of the wall. The greatest influence on the permeability of FDM parts is provided by the extrusion multiplier and the type of the internal filling of the wall: extrusion multiplier close to 1.00 (0.96–0.98) and diagonal internal filling of the wall tend to avoid permeability of the 3D-printed object since it results in the sealing of the pores in the.

### Scanning electron microscopy study

Scanning electron micrographs of FDM products were obtained using a Hitachi SU8000 field-emission scanning electron microscope (FE-SEM, Hitachi Ltd., Tokyo, Japan) at the Department of Structural Studies, Zelinsky Institute of Organic Chemistry in Moscow, Russia. Before the measurements, the samples were mounted on a 25 mm aluminum specimen stub and were fixed by a graphite adhesive tape. Metal coating with a thin film (7 nm) of gold/palladium alloy (60/40) was performed using the magnetron sputtering method. Images were acquired in the secondary electron mode at the 2–20 kV accelerating voltage and at the working distance of 8–10 mm. The morphology of the samples was studied taking into account possible influence of the metal coating on the surface.

### Experimental procedure for Suzuki-Miyaura and Heck reactions

#### Suzuki-Miyaura reaction

**Fig 7:**
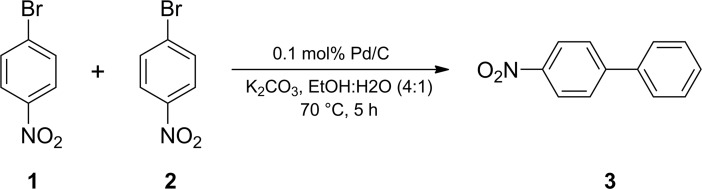
Suzuki-Miyaura reaction.

A test tube with a magnetic stirring bar was loaded with 1-bromo-4-nitrobenzene **1** (202 mg, 1.0 mmol, Sigma-Aldrich, 99%), phenylboronic acid **2** (146 mg, 1.2 mmol, Acros Organics, 99%), potassium carbonate (166 mg, 1.2 mmol, Acros Organics, >99%), 0.1 mol% Pd/C (10.6 mg, 1 μmol, Acros Organics), 1.6 mL of ethanol (Ferain, 95%) and 0.4 mL of H_2_O. The reaction mixture was stirred for 5 hours at 70°C and was analyzed by ^1^H NMR (Fig 7). The diameter of Pd nanoparticles at the commercial catalyst was 2.8 ± 0.7 nm, and the surface area of the catalyst was 1157 m^2^/g [49]. Commercially available reagents were used in the reaction.

#### Heck reaction

**Fig 8:**
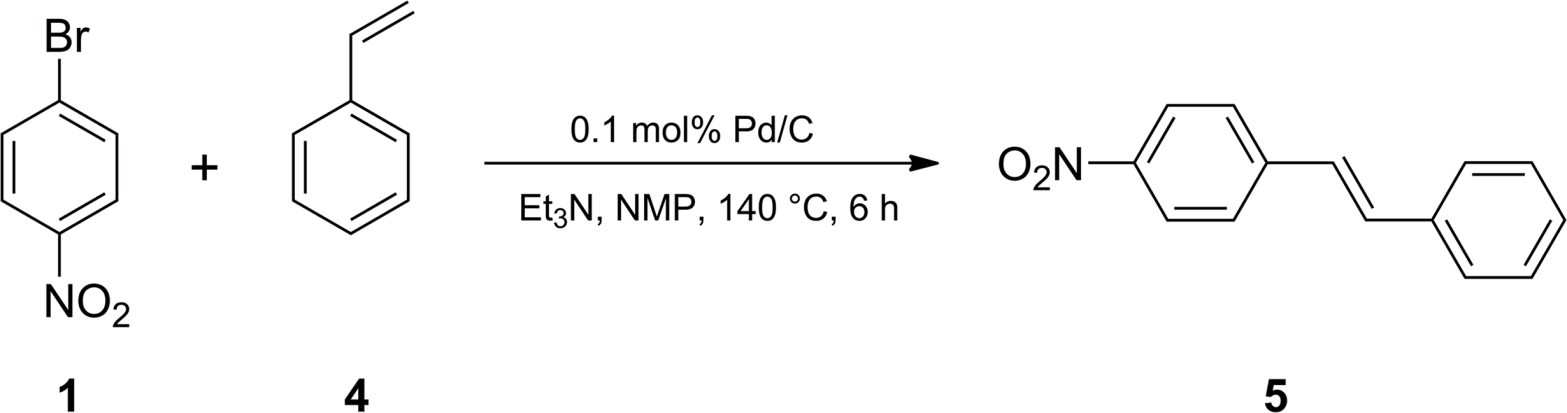
Heck reaction.

A test tube with a magnetic stirring bar was loaded with 1-bromo-4-nitrobenzene **1** (202 mg, 1.0 mmol, Sigma-Aldrich, 99%), styrene **4** (155 μL, 1.0 mmol, Acros Organics, 99%), triethylamine (167 μL, 1.2 mmol, Acros Organics, 99%), 0.1 mol% Pd/C (10.6 mg, 1 μmol, Acros Organics), and 2 mL of N-methyl-2-pyrrolidon (NMP, Carl Roth GmbH + Co. KG, >99%). The reaction mixture was stirred for 6 hours at 140°C and was analyzed by ^1^H NMR (Fig 8). Commercially available reagents were used in the reaction.

### NMR analysis of chemical reactions

NMR spectra were recorded on a Bruker DRX500 NMR spectrometer at room temperature. The residual proton (δ = 2.5) signal of DMSO-d6 was used as internal standard. 100 μL samples of the reaction mixture were taken and analyzed by ^1^H NMR (DMSO-d6, 500.1 MHz). The NMR spectra were processed using the MestReNova v6.0.2–5475 program package.

**Fig 9:**
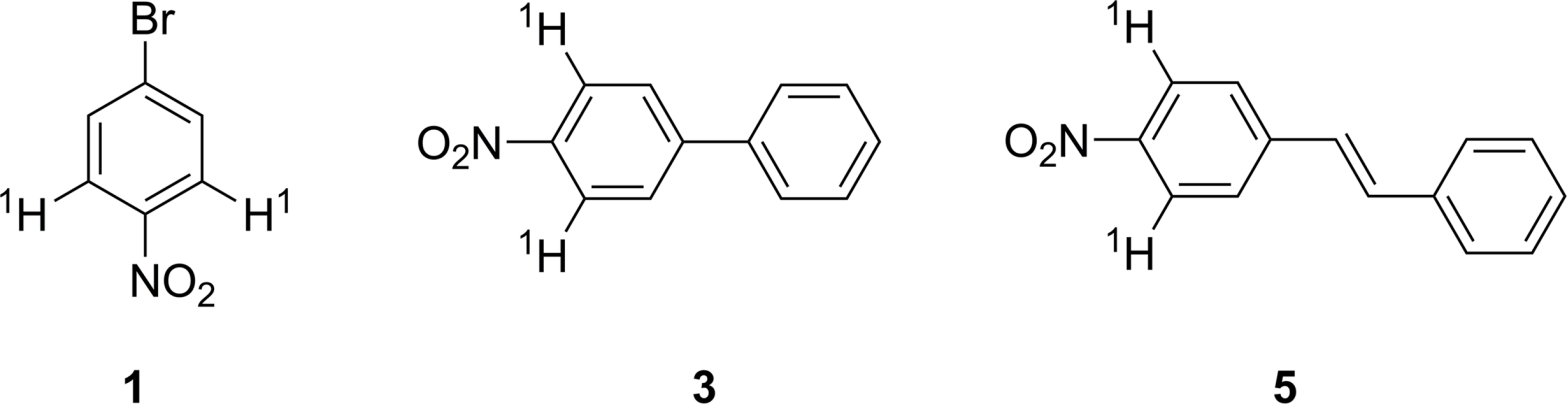
Protons in the molecules of reagents and products for conversion calculation by NMR study.

The conversion in the Suzuki-Miyaura reaction was calculated by analysis of the ratio of peak integrals of initial compounds and product (compound **1** (δ = 8.16, 2H) and compound **3** (δ = 8.30, 2H) (Fig 9)). The conversion in the Heck reaction was found by analysis of the ratio of peak integrals of initial compounds and product (compound **1** (δ = 8.16, 2H) and compound **5** (δ = 8.24, 2H)).

## Additional information

Experimental details, additional electron microscopy images and photographs are available in Supplementary Information.

## Supporting information

S1 Fig Cylindrical test tubes made of different materials. For all tubes the same extrusion multiplier (k = 0.98) and wall thickness (2 mm) were used.
(TIF)

S2 Fig PLA cylindrical test tubes manufactured with different extrusion temperatures. k = 0.98 for all tubes.
(TIF)

S3 Fig Dependence of product impermeability on wall thickness (PLA, k = 0.98). The values of the wall thickness (mm) are shown.
(TIF)

S4 Fig The vessels with composite geometric form made of different thermoplastic materials with different extrusion multipliers. (TIF)

S5 Fig SEM photography of the surface region of the FDM test tube manufactured with k = 0.85. (A) layered structure of the wall at small magnification, (B–F) interlayer space with micropores.
(TIF)

S6 Fig SEM photography of the surface region of the FDM test tube manufactured with k = 0.98. (A) layered structure of the wall at small magnification, (B–F) interlayer space with blind micropores.
(TIF)

S7 Fig The test objects, manufactured for this work by FDM method with different printing parameters. (A) vessels with different forms made of PLA; (B) test tubes manufactured with different values of the extrusion multiplier; (C) PLA test tubes manufactured with different extrusion temperatures and the same values of the extrusion multiplier; (D) PLA test tubes with different wall thickness; (E) test tubes made of different thermoplastic materials (Nylon-C is nylon filled by carbon fibers); (F) vessels with compositing geometric form made of different thermoplastic materials. See Experimental section for details.
(TIF)

S8 Fig The test tubes manufactured by Designer X Pro printer with different k-values. **(**A, B) impermeability tests; (C, D) differences in the internal structure of the wall; (E, F) differences in the quality of the external surface.
(TIF)

S9 Fig Bubbles distribution in “static” mode for vessels with different forms made of PLA. (A) cylinder, (B) cone, (C) sphere, (D) compound shape, (E) pyramid, (F) cube. Bubble distribution after internal pressure was dropped down to standard conditions.
(TIF)

S10 Fig Simple way for estimation of the volume of air passed through the vessel wall. (TIF)
