# Supplementary figures and images for: Improvement of quality of 3D printed objects by elimination of microscopic structural defects in fused deposition modeling
Source: PLoS One. 2018 Jun 7;13(6):e0198370. doi: 10.1371/journal.pone.0198370 (PMC5991691; doi:10.1371/journal.pone.0198370)

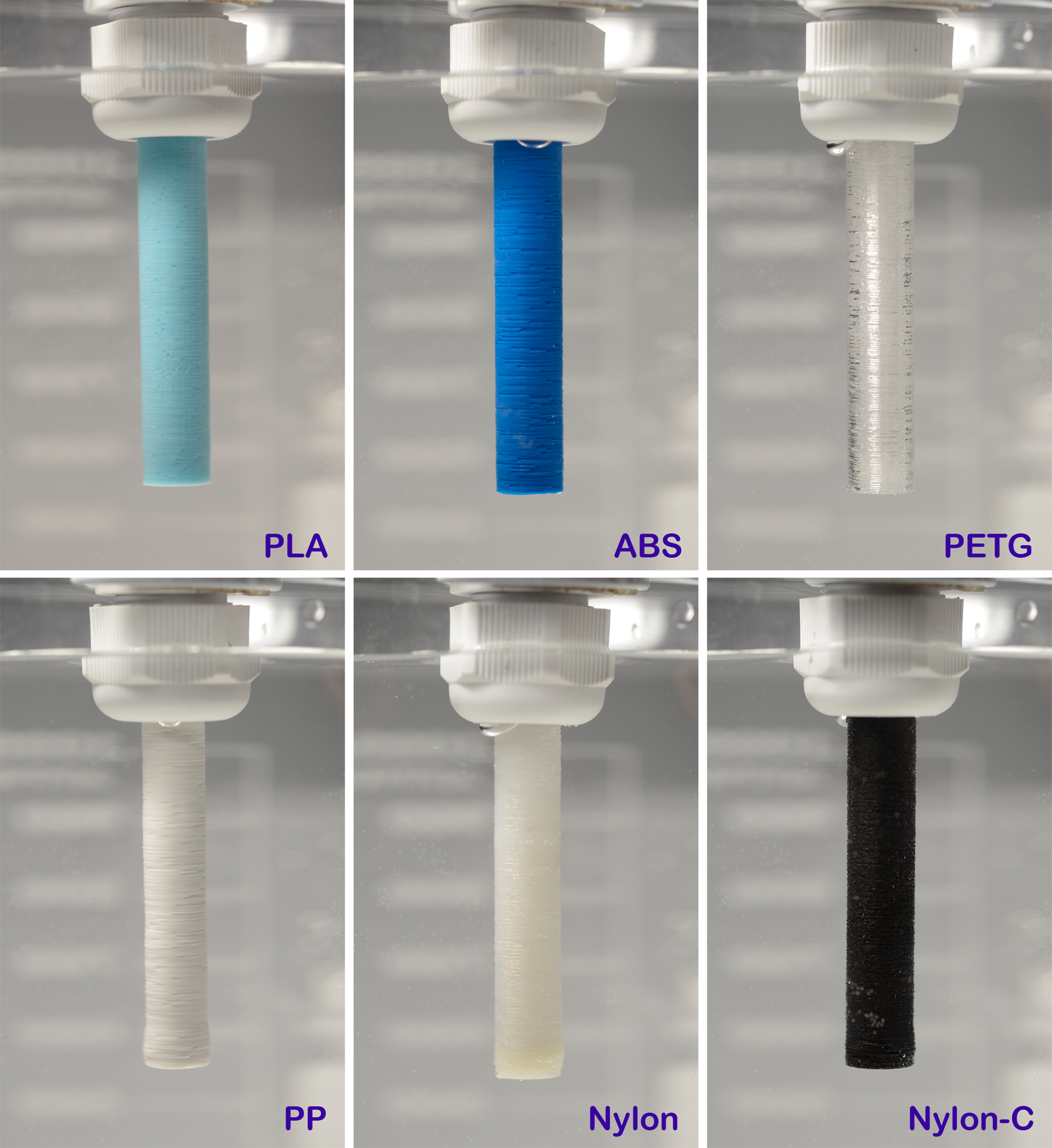

Supplement: S1 Fig — For all tubes the same extrusion multiplier (k = 0.98) and wall thickness (2 mm) were used. (TIF) [file pone.0198370.s001.tif]

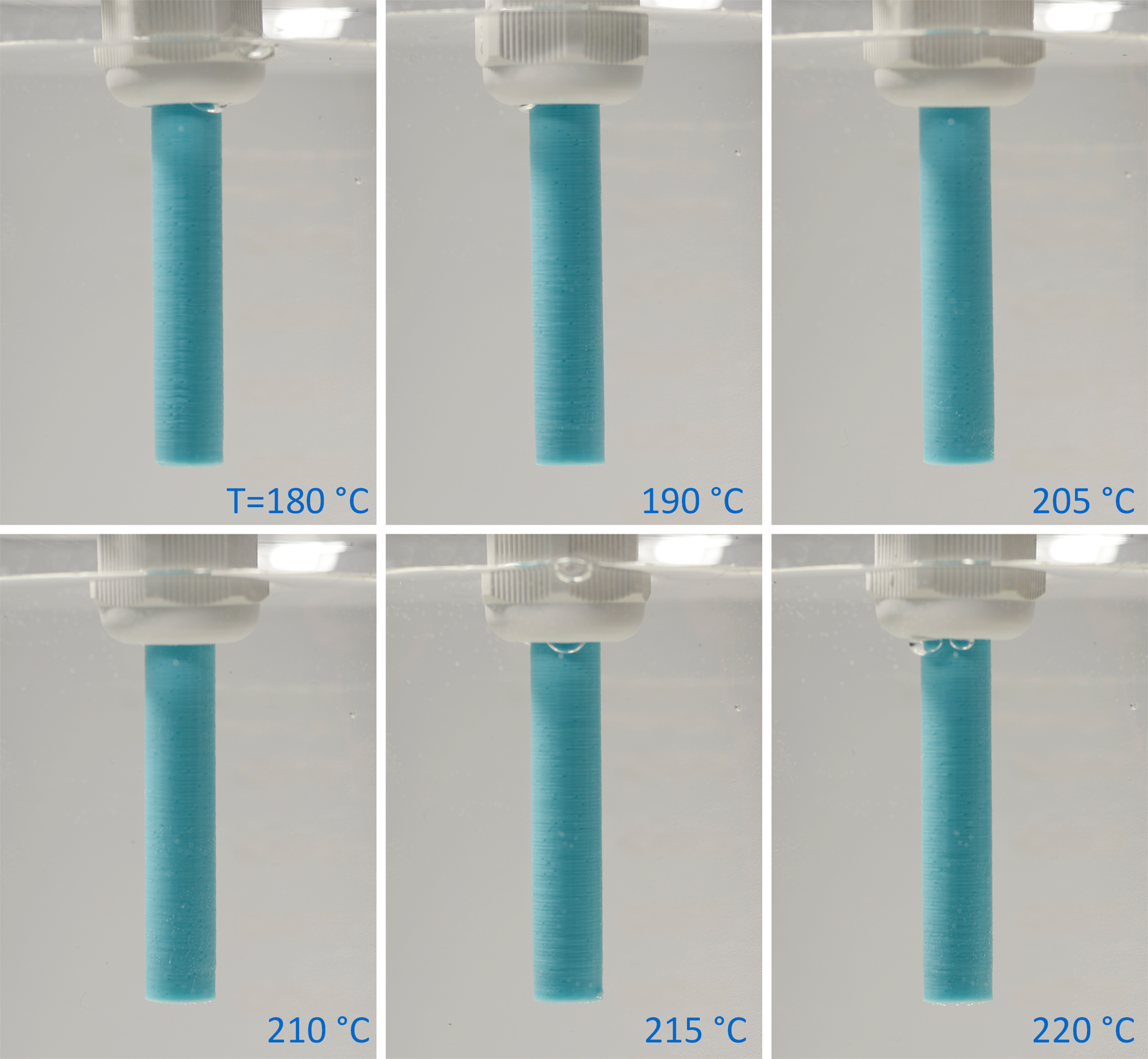

Supplement: S2 Fig — k = 0.98 for all tubes. (TIF) [file pone.0198370.s002.tif]

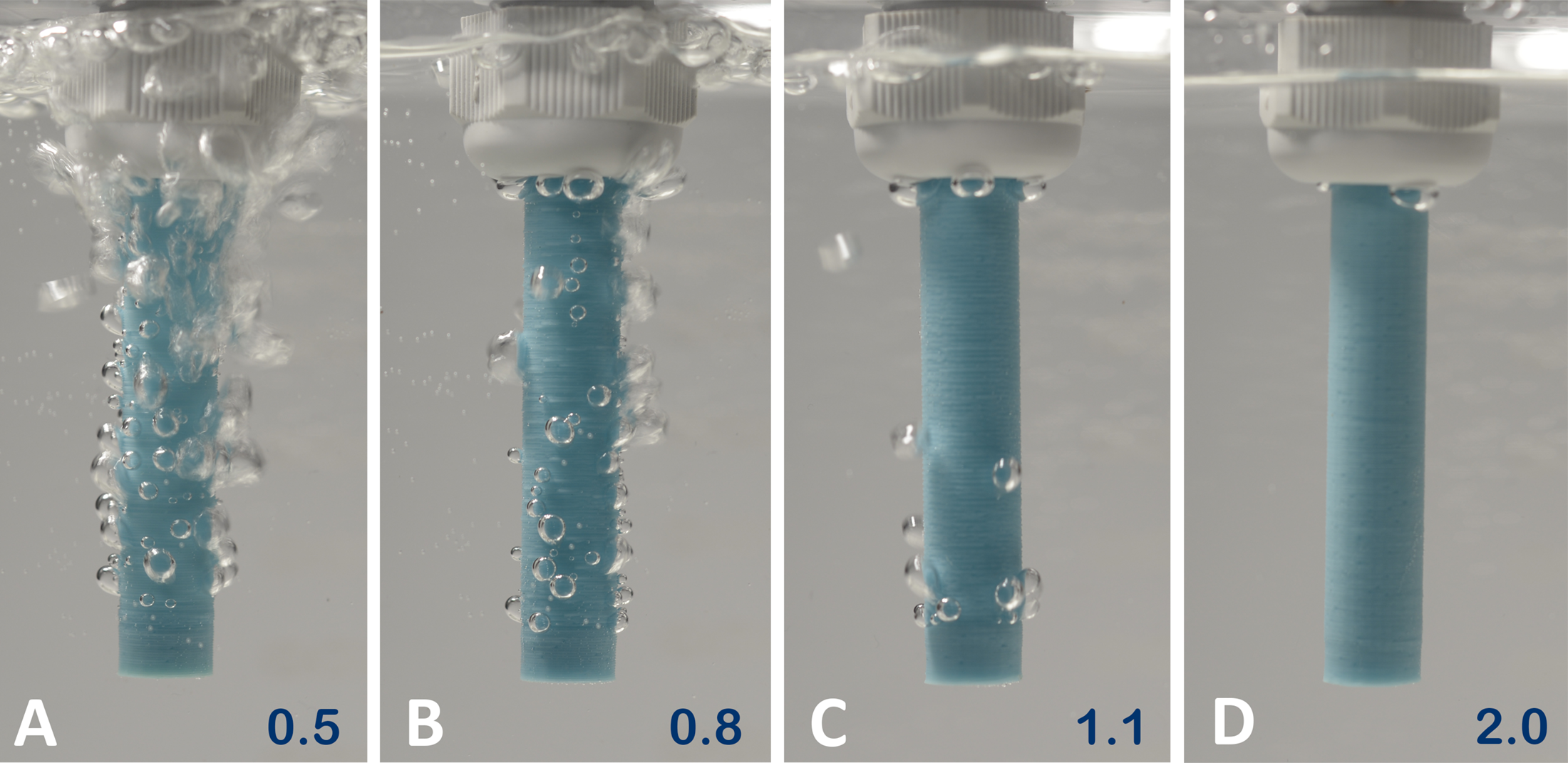

Supplement: S3 Fig — The values of the wall thickness (mm) are shown. (TIF) [file pone.0198370.s003.tif]

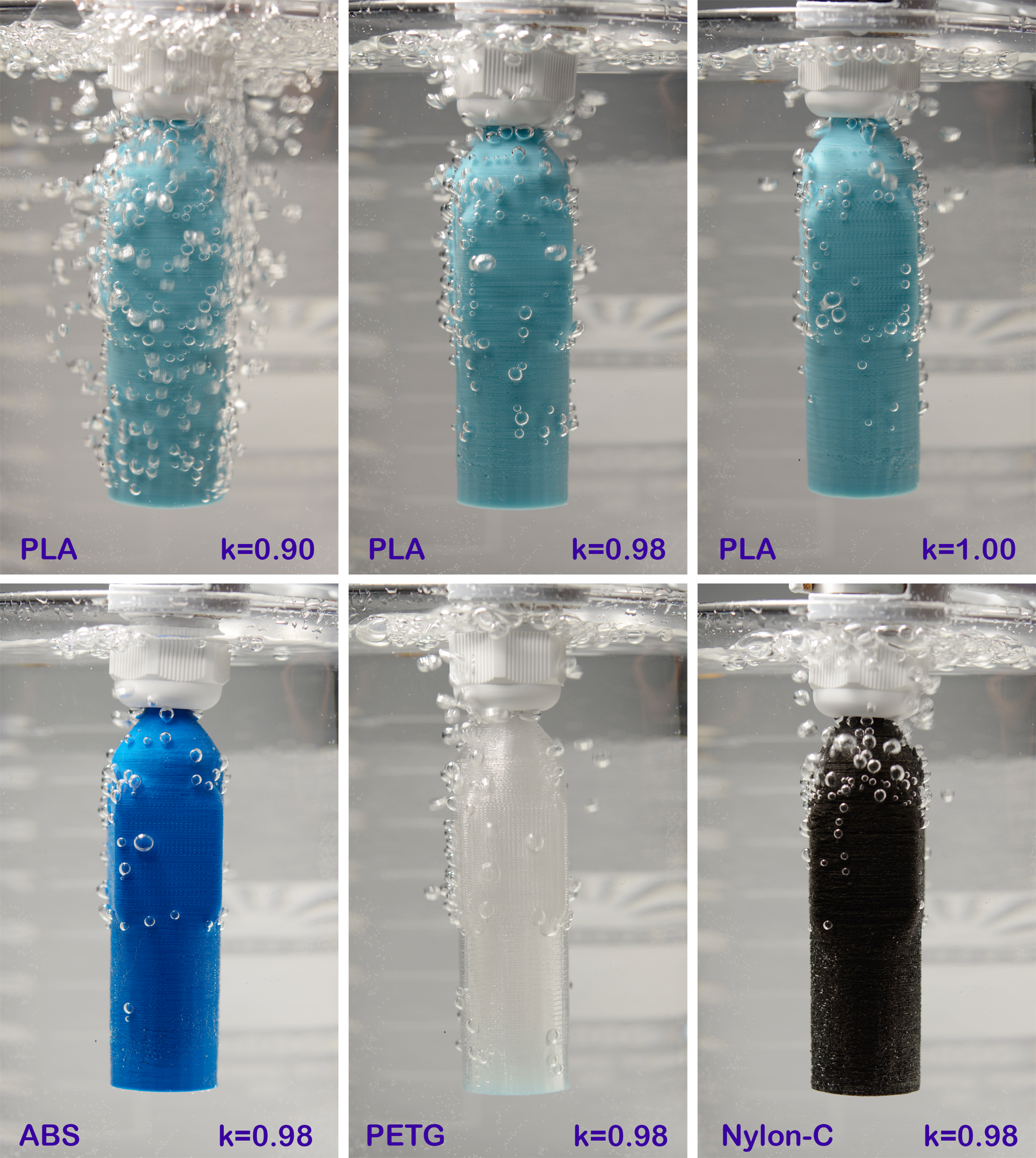

Supplement: S4 Fig — (TIF) [file pone.0198370.s004.tif]

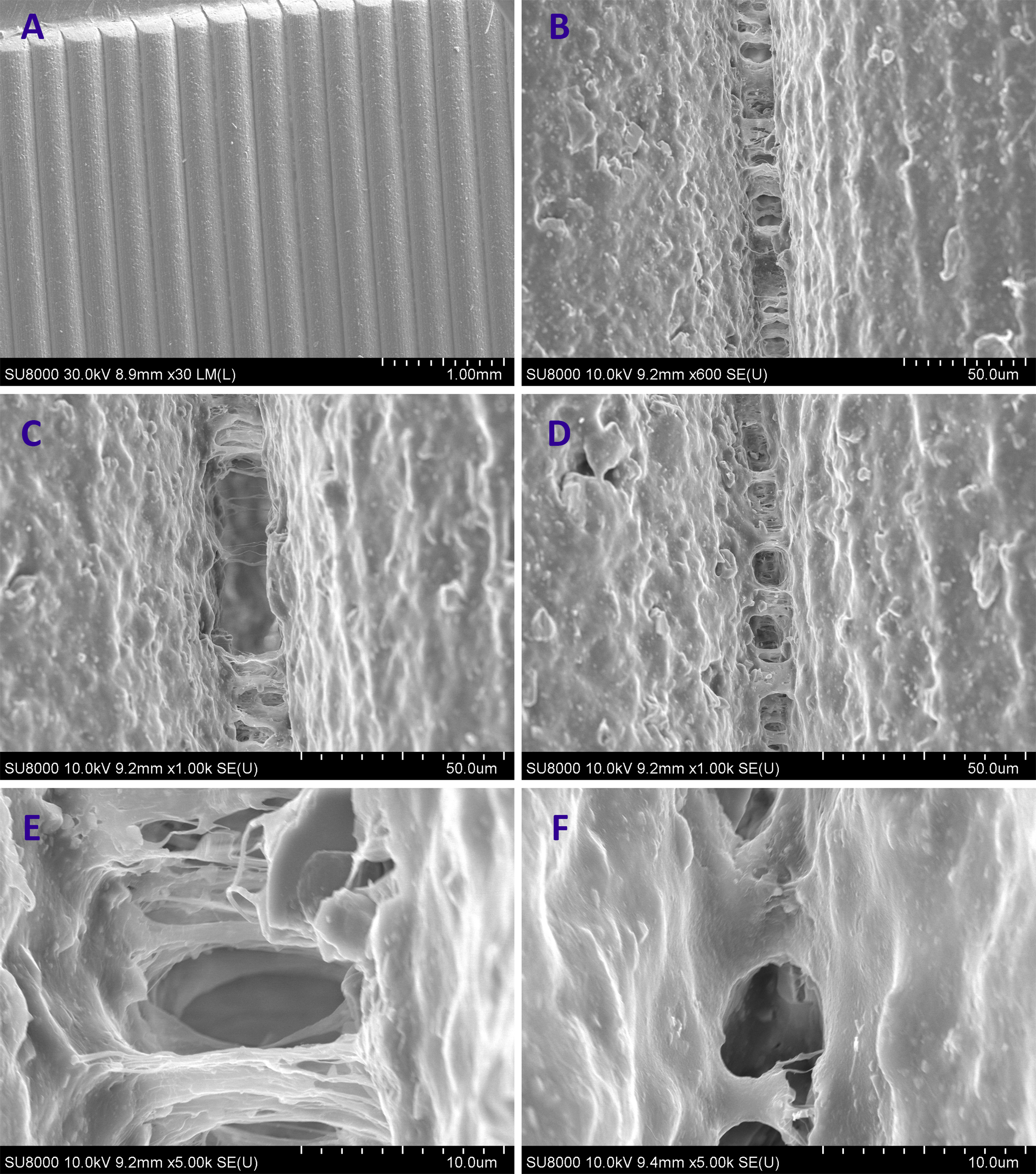

Supplement: S5 Fig — (A) layered structure of the wall at small magnification, (B–F) interlayer space with micropores. (TIF) [file pone.0198370.s005.tif]

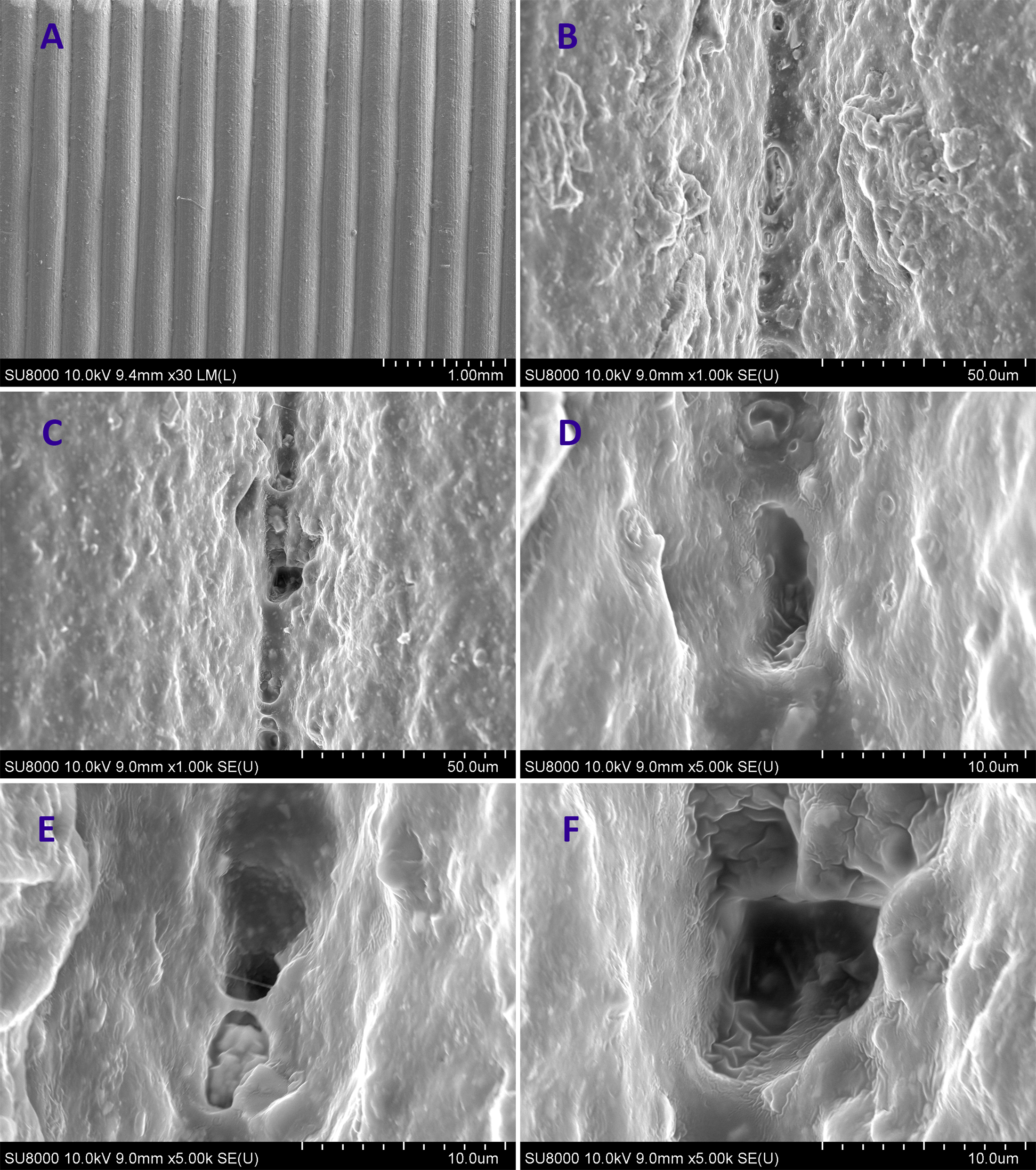

Supplement: S6 Fig — (A) layered structure of the wall at small magnification, (B–F) interlayer space with blind micropores. (TIF) [file pone.0198370.s006.tif]

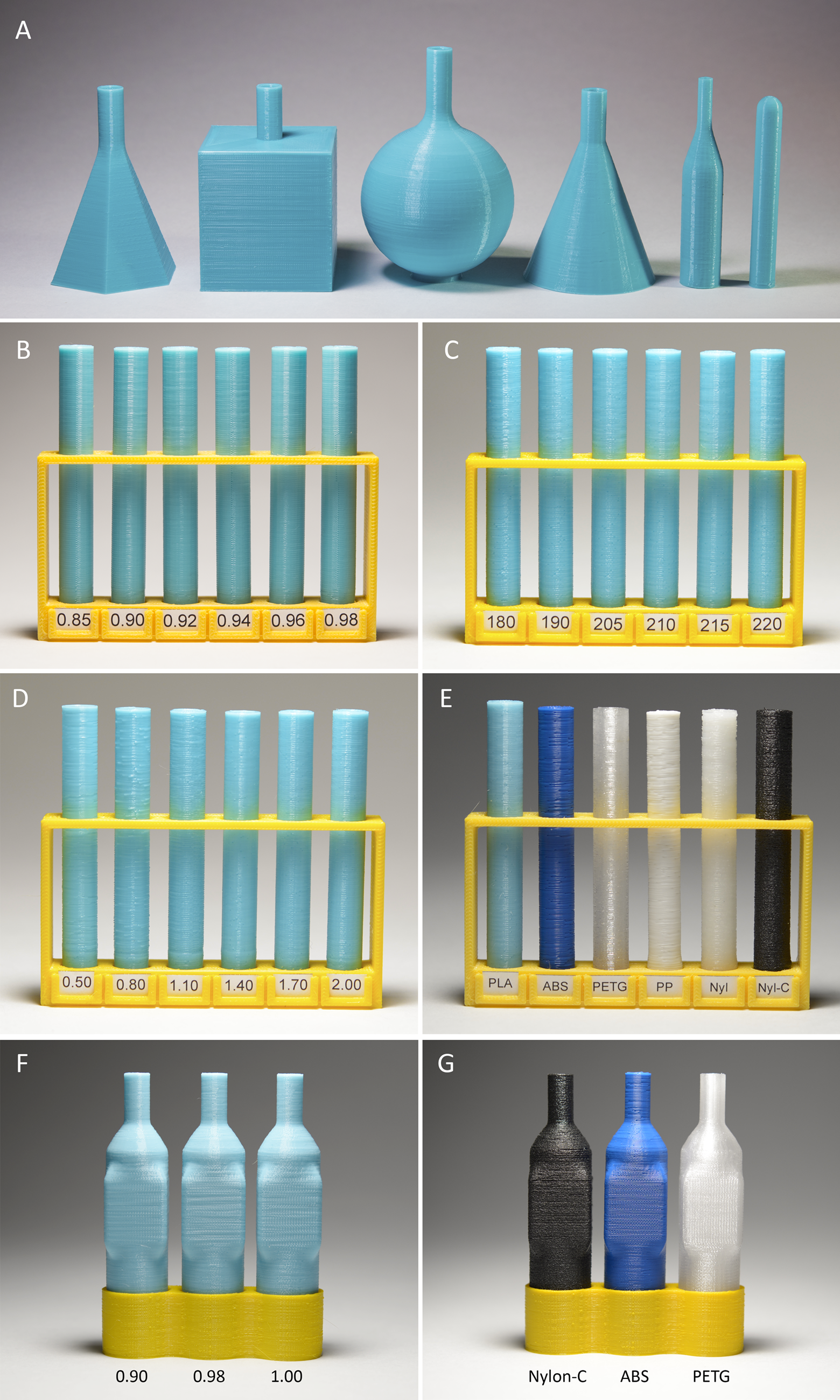

Supplement: S7 Fig — (A) vessels with different forms made of PLA; (B) test tubes manufactured with different values of the extrusion multiplier; (C) PLA test tubes manufactured with different extrusion temperatures and the same values of the extrusion multiplier; (D) PLA test tubes with different wall thickness; (E) test tubes made of different thermoplastic materials (Nylon-C is nylon filled by carbon fibers); (F) vessels with compositing geometric form made of different thermoplastic materials. See Experimental section for details. (TIF) [file pone.0198370.s007.tif]

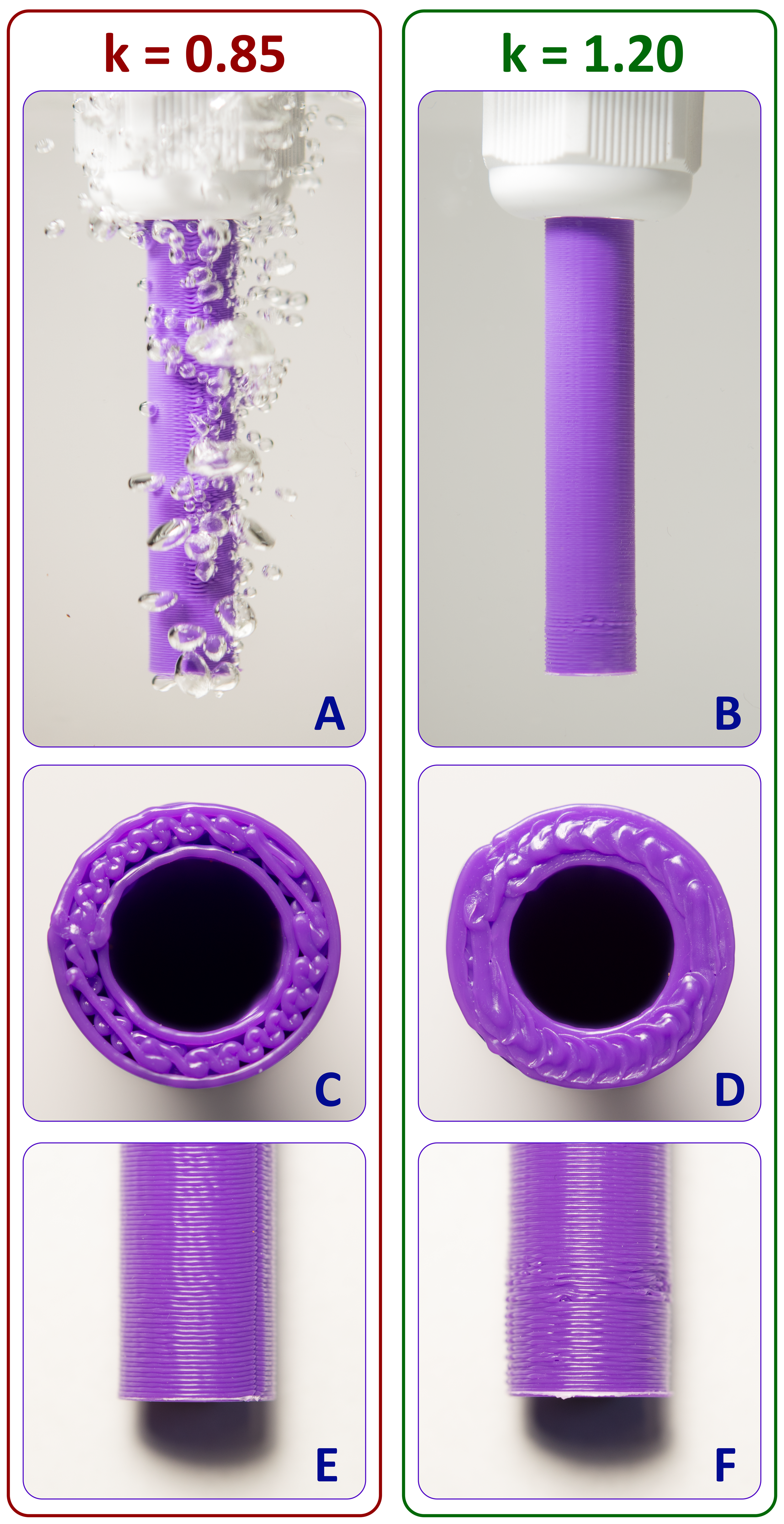

Supplement: S8 Fig — (A, B) impermeability tests; (C, D) differences in the internal structure of the wall; (E, F) differences in the quality of the external surface. (TIF) [file pone.0198370.s008.tif]

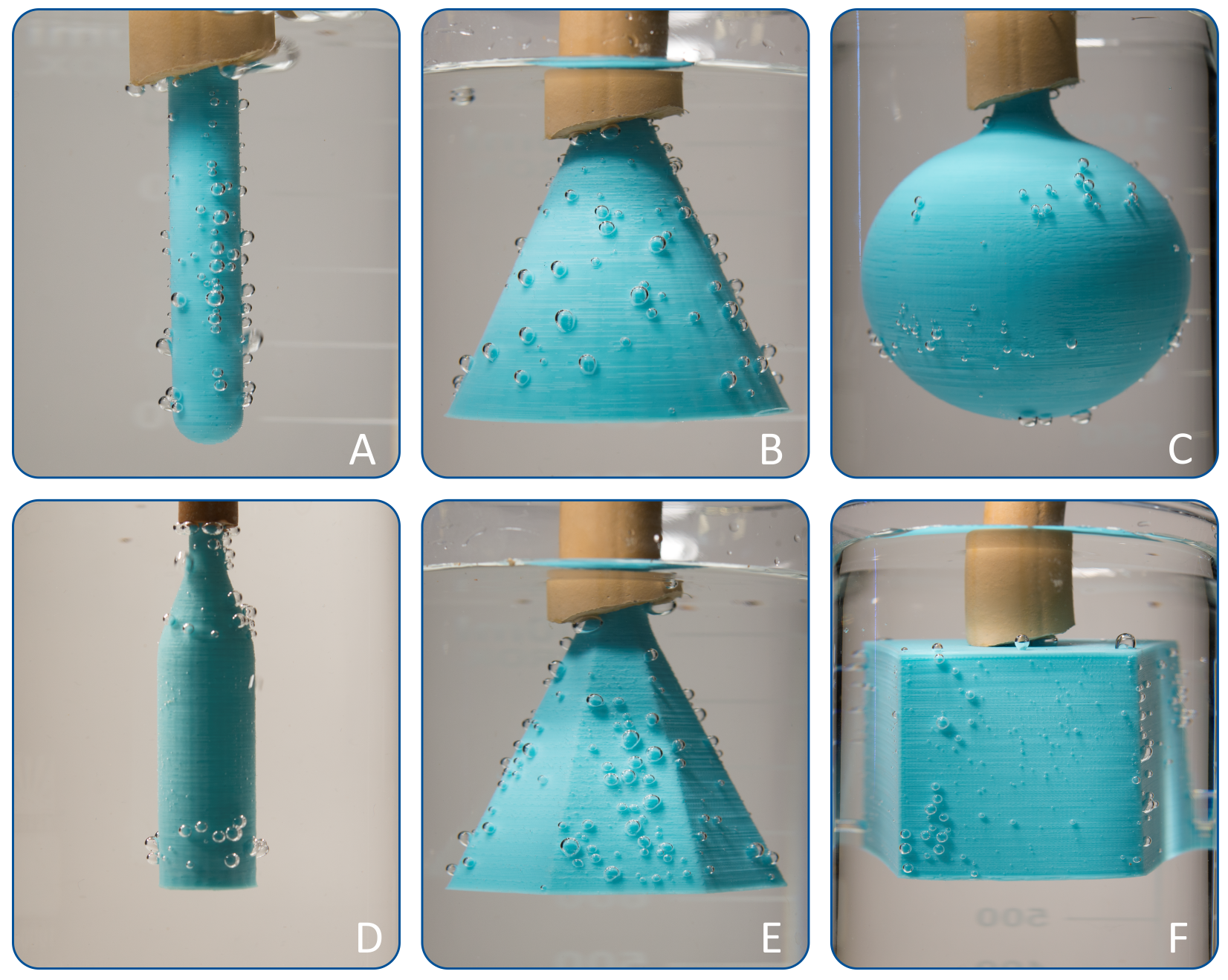

Supplement: S9 Fig — (A) cylinder, (B) cone, (C) sphere, (D) compound shape, (E) pyramid, (F) cube. Bubble distribution after internal pressure was dropped down to standard conditions. (TIF) [file pone.0198370.s009.tif]

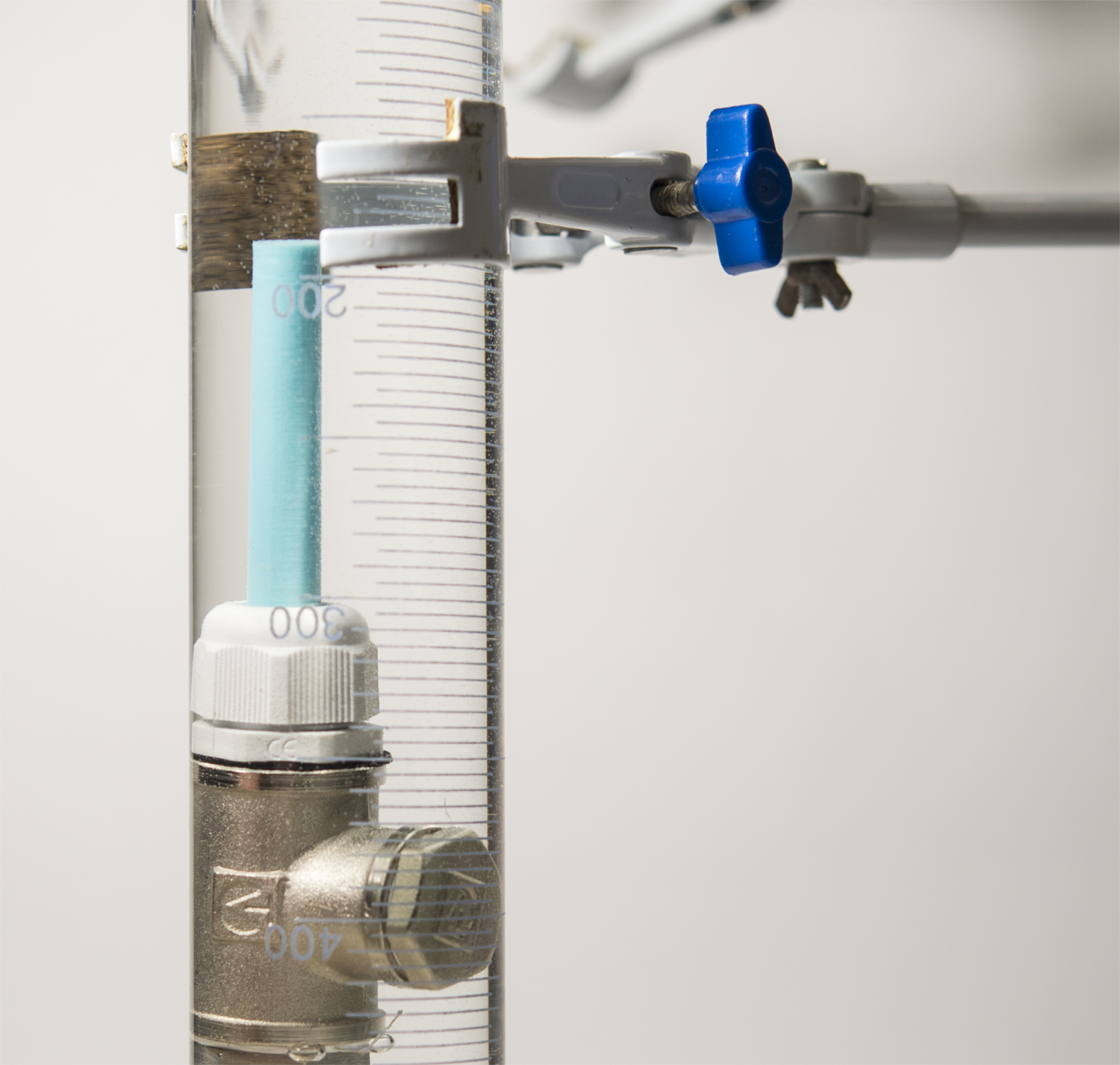

Supplement: S10 Fig — (TIF) [file pone.0198370.s010.tif]
